# Contributions to Operative and Mechanical Dentistry

**Published:** 1844-12

**Authors:** W. H. Elliot

**Affiliations:** Fellow of the American Society of Dental Surgeons.


THE AMERICAN

### JOURNAL OF DENTAL SCIENCE.

Vol. V.] DECEMBER, 1844. [No. 2.

### ARTICLE I.

*Contributions to Operative and Mechanical Dentistry.* By W. H. Elliot, D. D. S., Fellow of the American Society of Dental Surgeons.

No. 4.

### UPON THE CONSTRUCTION OF ARTIFICIAL TEETH UPON PLATES.

It is the duty of the surgeon dentist, when called upon to exercise his skill in the mechanical department, to decide, after a deliberate examination, upon such a course as shall secure to his patient the fullest benefit of the dental art. The objections generally urged by the patient to the plan proposed, are of no account, compared with the great advantages to be derived from a thorough operation. It is true that he may be advised of the evils that a partial course will bring upon him, and by that means, although he will not be satisfied, he may be hindered from uttering a word of complaint; and still, as often as he opens his mouth, the work will speak for itself, in a language too eloquent for the credit of him who constructed it. Not only the welfare of his patient, but his own reputation, demands a strict adherence to the correct principles of this branch of dental surgery. And should all his arguments fail to produce the desired effect upon the mind of his patient, he should respectfully yet firmly decline pursuing a course which would be at once productive of injury to his employer and disgraceful to himself. If the pecuniary circumstances of his patient be offered as an objection, it may be his duty to divide the profit, but in no instance to divide the operation. It is this unfortunate willingness to be governed by the opinions of the whimsical and superstitious, that so completely blends the practice of the scientific surgeon dentist with that of the most contemptible empiric, and which effectually entails upon the profession its poverty of usefulness and of public confidence. He who desires the advancement of dental surgery, should have fixed principles, and in all cases be governed by them: for no profession can rise on the scale of usefulness and respectability, while its members are constantly taking backward strides.

As the Journal has already furnished to its readers the fullest and most correct treatise on mechanical dentistry ever written,* the writer does not deem it necessary to enter into all the particulars of the *modus operandi*, except upon those points in his practice differing from the methods heretofore recommended.

Premising that sufficient time has elapsed after the removal of the teeth, for the gums and alveolus to have become permanently formed, and that the remaining teeth have been put in a perfect state of health, the dentist may proceed at once to take the cast. To do this successfully, he should be supplied with a large number of wax boxes of different forms and sizes; they should be made of a single piece of German or pure silver-plate, and formed by being *swedged* between a model and counter model, in the same manner that a gum plate is fitted to the mouth; and to prevent the wax from pressing away the soft parts, the box should approach the gums nearest at its edges. In pressing the wax upon the mouth, the operator should see that the pressure be given to all parts of it equally; this is best done by placing a finger upon each side of the box, the handle only being useful in withdrawing the cast from the mouth. The cooling of the wax may be facilitated by directing the patient to inhale through the mouth and exhale through the nasal passage, and when it has become sufficiently hardened, the whole may be passed out of the mouth between the blades of an instrument similar to the *speculum uteri* of the *accoucheur*, without coming in contact with any thing after it leaves the gums. The use of this instrument secures the cast from any accident that might

* Brown's Treatise.

otherwise happen to it on its passage out of the mouth. The cast may now be put into an iron box, with the impression upward, and the plaster poured upon it. This box should be of sufficient capacity to receive the largest cast, and should have partitions fitted to it at different places, so that its size may be altered. It should also be much smaller within at the bottom than at the top, so that it may give to the plaster model the required form.

The smaller the proportion of water used in mixing the plaster, the stronger will be the model when dried.

Care should be taken that each depression of the wax be filled with the plaster. This should be done carefully with a small pointed spatula.

At this stage of the process, the dental artist will find use for a number of short knives, curved in different directions, for the purpose of trimming the model.

A leaden plate of the usual thickness of gold may now be fitted to the model for a pattern, and its edge traced upon the plaster with a pencil, except where it comes in contact with the natural teeth, then, in place of the pencil mark, a little groove may be cut, equal in depth to the thickness of the plate. The plaster may now be scraped or cut away to the distance of a line upon the plate side of the groove. The object of making the groove instead of cutting away the plaster at once, is to get at the exact depth, which it would be difficult to do otherwise. The groove must vary a little in depth, it being necessary to cut it deeper over the softer parts. This is the only method of bringing down the edge of the plate to a closer fit upon the gum that can be adopted with safety, it being impossible to turn the edge after it is fitted, without altering its whole form.

The plaster model, after being about half-dried and laid in a cool place for half an hour, is fit for use.

The results of numerous experiments which have been instituted by the writer, upon the construction of models and counter models, have led him to the following conclusions:

1st. That the best material for a metallic model, on account of its solidity and fusibility, is a compound of one part of copper and twelve parts of pure tin.

2d. That lead, on account of its softness, and particularly on account of its disposition to part with its caloric when brought in contact with other bodies, together with the fact of its being a bad conductor, is not surpassed as material for counter models.

3d. That the ladles must have flat bottoms, so that they will remain firm while under the force of the hammer.

4th. That their sides must flare to an angle of about forty-five degrees from a perpendicular.

And lastly, that the plaster model, when plunged, must be cold and a little damp, so that it may receive with greater facility the caloric of the lead.

The lead should be fused over a slow fire, so that the ladle and metal may be heated alike, and when perfectly melted, it may be withdrawn from the furnace, and rested upon something that will not have a tendency to cool the ladle. In this position it must be stirred briskly, to prevent it from stiffening at the sides ; and when so far congealed that it will not return to an equilibrium, it is fit to receive the plaster model, which must be plunged at once as near the bottom as possible. The lead immediately becomes solid between the plaster model and the bottom of the ladle, and its loss of volume by shrinkage is supplied from the large mass of fused metal above surrounding the model. At this moment, the two apparently opposite qualities of the lead, mentioned above, act in unison for the perfection of the work. The coating of solid lead which is instantly formed upon the plaster, gives off its caloric faster than it receives it from the surrounding metal, and therefore remains perfect. The former of these qualities is owing rather to its weight, which brings it in closer contact with bodies, than to any constitutional property of the metal.

After the plaster has been perfectly removed, the counter model should be coated with lamp-black, by being held over a smoking lamp; no other precaution is necessary to prevent the union of the metals, provided the composition of the model, when poured, be no hotter than is requisite to render it fluid. A strong wrought or cast iron band may now be placed upon the counter model, surrounding the depression made by the plaster. This should be filled with the composition of the model, and allowed to remain upon it while in use. The counter model should also remain in the ladle to prevent it from spreading.

For fitting the plate it is necessary to be provided with a pointed mallet and a wooden stake, having a shallow depression upon the surface of it, corresponding somewhat with a counter model. After it has been partially fitted by this means, it may be placed upon the counter model, and forced into it with a wooden set and hammer. The plate should then be oiled and placed between the two metals, for the purpose of forming it. A little conical block, about four inches in length, placed upon the model when in use, will protect it from injury by the blow. The sledge should weigh at least twelve pounds, for it not only requires great force to strike up a large plate, but it wants just that kind of jar which is given by a heavy hammer to adjust the model as it passes into the lead. It hardly need be mentioned, with regard to the use of a smith's vice for this purpose, that the jaws of that instrument do not allow the metals to adjust themselves, and therefore a perfect fit of the plate cannot be obtained.

The plate should be thoroughly annealed after it has been accurately fitted, and then placed between the metals for the last time, otherwise it will spring considerably out of true, during the process of soldering. The teeth may now be selected, and fastened temporarily upon the plate, by means of a cement composed of three parts of Burgundy pitch and one part of white wax, rendered sufficiently hard by he addi tion of a little rosin. This cement may be made to adhere with greater tenacity, by heating the plate and teeth, and by coating with it those parts which are afterwards to receive it in larger quantities. In applying the cement, small portions of it must be laid against the back of the tooth, and melted down with an iron previously heated over a lamp, so as to connect the tooth and plate.

At this stage of the process, the presence of the patient is necessary, for the arranging or antagonising of the teeth, for in that lies the great secret of rendering them useful; and yet, as important as it is, it is impossible to lay down rules by which the student may in all cases be governed.

In case the entire upper set is to be supplied, and retained in its place by the pressure of the atmosphere, and there remains only the *incisores*, *cuspidati* and two or four *bicuspides* in the lower jaw, and it be not thought expedient to supply the loss of the lower *molares*, instead of placing *cuspidati* upon the plate, their place should be occupied by teeth bearing an external resemblance to *cuspidati*, yet in reality *bicuspides.* This gives to the set two extra small grinders, and renders it doubly serviceable. But if, in addition to the want of the *molares* of the lower jaw, the *bicuspides* be so much inclined inward that the artificial substitutes of the upper jaw cannot be made to meet them without destroying the symmetry of the countenance, (an accident common in second dentition,) an entire set of single teeth may be chosen, using *cuspidati* for *bicuspides* and *molares*, and these arranged upon the plate without regard to any thing but beauty and elegance, and after they are permanently soldered, a second row of masticating blocks may be placed upon the plate, one directly over each irregular double tooth below, and soldered securely as the first. These little blocks are best made from *bicuspides* and *molares.* They should be as short as circumstances will permit, and perfectly fitted to their places by grinding.

The value of this method of arranging the teeth, in certain cases, is incalculable, for while the outer row restores the countenance to its natural fullness and beauty, the inner row is made vastly more serviceable by being shorter and placed nearer the middle of the plate.

A small instrument resembling a light pair of curved forceps, will be found useful for holding the teeth upon the stone, the beaks of which should not, like the forceps, approach each other at the points; but they should be hollowed longitudinally, and lined with copper.

It should be remembered that by making the inner points of the superior artificial grinders considerably longer than the external, pressure upon them always inclines them inwards, the direct effect of which is to render the plate permanent. Nor should the patient be allowed to forget that the utility of artificial teeth depends more upon the skill he acquires in the use of them, than upon their attachment to the natural teeth or gums.

It not unfrequently happens that the patient is unable, even in common conversation, to keep the gums of the upper jaw hid from view, in consequence of a peculiar projection of the *alveolus*, both forward and downward ; if, in such a case, a "*suction*" plate be required, it is impossible to cover the external gum without rendering the plate liable to exposure whenever the mouth is opened. This difficulty may be remedied by bringing the plate as high upon the labial gum as practicable, and then by cutting away all that portion of the plate which is exposed by raising the lip, thereby forming an opening through which the gum may appear. The form and size of the opening may be determined as follows : after the teeth are fastened temporarily upon the plate, the whole may be placed in the mouth, and the patient directed to raise the lip as high as possible, the contour of which may then be traced upon the plate, from the first *bicuspis* upon one side to the corresponding tooth upon the other; the outlines of the upper ends of the teeth may be traced in the same manner and to the same points, and then all that portion of the plate between the two lines may be removed. The edge of the plate along the under side of the opening must be filed thin, and bent to a closer fit upon the gum, so as to give to the teeth the appearance of projecting therefrom, as in case of a partial set with clasps.

In arranging the teeth upon the plate, it is necessary to allow something for the contraction of the solder in cooling, otherwise the circle will be so narrow at the grinding surface as to destroy the beauty of the work if not its utility. This is not so apparent in short teeth, but long teeth are more or less liable to be moved inward by the solder while cooling.

When the teeth are adjusted upon the plate, a thin iron or copper band may be procured, and bent into a form corresponding with that of the work to be soldered. This band may then be laid upon some level surface, and a mixture of one part of gypsum and two parts of talck, or, in the absence of talck, the same proportions of gypsum and common sand, reduced to a proper consistency with cold water, may be poured into it in sufficient quantity to form a temporary bottom about one line in thickness ; the concavity of the plate may be filled with the same mixture and the whole placed in the band, with the teeth upward. The space between the enamelled surfaces of the teeth and band, which should be about two lines in width, may also be filled to a level with the cutting edges of the teeth.

After the teeth have been supplied with backs, their ends should be covered with the mixture to prevent them from rising from their several positions, and also to protect them from the heat of the blow-pipe.

The punch for making the holes through the backs should be double, on the principle of the depthing tool of the watch-maker, and it may be set by separating the dies until each of the hollow ones will receive a rivet of the tooth. If the double punch cannot be procured, the hollow pointed dividers and single punch will do as well; the dividers should be set so that each point will receive a rivet of the tooth, and then by pressing them upon the backs two small rings will appear, indicating the exact position for the holes.

The backs of the teeth should be just sufficiently raised in the centre to bring the edges to a perfect fit upon the tooth; if raised more than that, the rivets are weakened proportionably.

For fastening the backs upon the teeth, it is only necessary to file the rivets nearly level with the plate, and then split them down with a strong knife, so as to spread them in every direction. The riveting hammer not only destroys many teeth directly, but is one cause of their cracking under the blow-pipe.

The blow-pipe which has been in use by the writer for the last four or five years, and which he described, three years since, in a letter to Dr. Brown of New York, is sufficiently powerful to accomplish the object in as short a time as the safety of the work will allow, without previously heating it, or encumbering it with charcoal. This instrument has three large wicks, each one inch in diameter. It also has three pipes so arranged as to throw the flames into a focus at a proper distance from the wicks. The wicks are supplied with oil through a small stop-cock, from a reservoir situated considerably above them. By means of the stop-cock the oil is let into the wicks just fast enough to give the greatest possible heat. The pipes are supplied with air from a bellows, propelled by the foot. While using this instrument both hands of the artist are at liberty to manage the work, as with the self-acting blow-pipe.

It is frequently necessary to alter the grinding surfaces of the teeth after they are soldered, for the purpose of bringing them to a more perfect bearing upon the opposite set. This may be done by means of a small leaden wheel, one inch in diameter, and two lines in thickness, coated with emory and oil, and running upon the end of a lathe spindle.

To learn where the teeth of one set interfere with those of the opposite, those parts which are supposed to touch may be covered with a thin sheet of wax, and the patient directed to close the teeth upon it; the appearance of the wax will indicate correctly the several points of contact.

